# Wire Marking Results in a Small but Significant Reduction in Avian Mortality at Power Lines: A BACI Designed Study

**DOI:** 10.1371/journal.pone.0032569

**Published:** 2012-03-01

**Authors:** Rafael Barrientos, Carlos Ponce, Carlos Palacín, Carlos A. Martín, Beatriz Martín, Juan Carlos Alonso

**Affiliations:** Departamento de Ecología Evolutiva, Museo Nacional de Ciencias Naturales (CSIC), Madrid, Spain; University of Plymouth, United Kingdom

## Abstract

**Background:**

Collision with electric power lines is a conservation problem for many bird species. Although the implementation of flight diverters is rapidly increasing, few well-designed studies supporting the effectiveness of this costly conservation measure have been published.

**Methodology/Principal Findings:**

We provide information on the largest worldwide marking experiment to date, including carcass searches at 35 (15 experimental, 20 control) power lines totalling 72.5 km, at both transmission (220 kV) and distribution (15 kV–45 kV) lines. We found carcasses of 45 species, 19 of conservation concern. Numbers of carcasses found were corrected to account for carcass losses due to removal by scavengers or being overlooked by researchers, resulting in an estimated collision rate of 8.2 collisions per km per month. We observed a small (9.6%) but significant decrease in the number of casualties after line marking compared to before line marking in experimental lines. This was not observed in control lines. We found no influence of either marker size (large *vs.* small spirals, sample of distribution lines only) or power line type (transmission *vs.* distribution, sample of large spirals only) on the collision rate when we analyzed all species together. However, great bustard mortality was slightly lower when lines were marked with large spirals and in transmission lines after marking.

**Conclusions:**

Our results confirm the overall effectiveness of wire marking as a way to reduce, but not eliminate, bird collisions with power lines. If raw field data are not corrected by carcass losses due to scavengers and missed observations, findings may be biased. The high cost of this conservation measure suggests a need for more studies to improve its application, including wire marking with non-visual devices. Our findings suggest that different species may respond differently to marking, implying that species-specific patterns should be explored, at least for species of conservation concern.

## Introduction

Bird collisions with electric power lines have raised conservation concerns since the early 1900s, but it was not until the 1970s that biologists and engineers began to realize the extent of this problem [1], [2]. Today the number of power lines is increasing worldwide at an annual rate of approximately 5% [3]. Mortality from collisions with power lines and other electric utility structures has been documented for some 350 bird species [4]. However, until a cumulative impacts assessment of power line mortality is conducted, the real level of mortality will remain uncertain [5]. Only some crude estimates of the importance of the problem, all of them based on extrapolations, are available. For example, in the Netherlands it has been found that bird collisions with power lines may cause one million deaths per year [6]. In the United States [5], it is estimated that power lines may kill up to 175 million birds annually, and it is estimated that bird collisions with power structures, including transmission (≥70 kV, usually with ground-wire and wires at more than one height) and distribution (<70 kV, commonly without ground-wire and all the wires at the same height) lines, could approach one billion avian fatalities per year worldwide [7]. Fortunately, these values are probably overestimated since most of the studies are usually carried out on power lines that cause an important number of fatalities. Nevertheless, these figures allow conservationists to speculate that mortality due to collisions with power lines represents a serious threat for population viability in many species, at least in those that undergo higher collision risks, and that this threat is not equal for all species. Indeed, birds with low manoeuvrability, i.e., those with high wing loading and low aspect, such as bustards, pelicans, waterfowl, cranes, storks, and grouse, are among the species most likely to collide with power lines [2], [8]. Species with narrow visual fields are also at high collision risk as they do not see the wires [9], [10]. Despite this potentially important conservation problem, few studies have analyzed in detail how these losses affect population trends. For instance, it has been estimated that collision-related losses might equal up to 90% of the annual number of grouse harvested by hunting in Norway [11]. Based on ring-recovery data [12], it has been assessed that 25% of juveniles and 6% of adult white storks (*Ciconia ciconia*) die annually in Switzerland due to power lines (although these data also include electrocutions). It has also been estimated that 30% of Denham's bustards (*Neotis denhami*) die annually by collisions with power lines in South Africa [13].

Researchers and managers have used several methods to reduce collisions, including the removal of the static wire [14], [15]. However, the most popular measure has been the attachment of spirals, plates, swivels, or spheres (collectively known as *bird flight diverters*) to the static wire in order to increase visibility [3], [16], [17], [18]. While a recent review concluded that marking static wires reduces the overall number of bird casualties at power lines, it also called attention to the fact that there are a surprisingly small number of well-designed, peer-reviewed studies to support this [19]. Furthermore, there remain many gaps in the research in this area, with several important details still unresolved; for example, the comparative effectiveness of various currently available marker types [19]. To confirm diverter effectiveness, and to study all details of this conservation measure in depth is especially important because despite the high costs of wire marking (e.g., 1,100–2,600 US$ per marked kilometre in South Africa, [20]; 6,000€ in Spain; [21]), the application of this conservation measure is rapidly increasing worldwide.

As stated above, it has been shown that the presence of flight diverters was associated with a decrease in bird collisions [19]. However, the large differences in wire-marking techniques constrained the ability to evaluate potential differences among methods (e.g., different performance based on diverter traits) in that review. To complement such an approach, in the present study we designed the largest field experiment to date, to investigate: (i) the effectiveness of wire marking in reducing collisions; and the roles of (ii) power line type (transmission *vs.* distribution), and (iii) spiral size on marking effectiveness. We expected that: (i) the attachment of spirals would reduce bird mortality [19]; (ii) the effectiveness of marking would be higher in transmission lines because power line type influences the frequency of reactions to marked spans [22]. Morkill & Anderson [22] found that whooping cranes (*Grus americana*) reacted more than expected to transmission lines (345 kV, 27 m high) whereas the opposite was true in distribution lines (69 kV, 12 m high). It is worth noting that transmission lines in our study accumulate a larger number of collisions of those groups of birds especially prone to collision, such as bustards, storks or waterfowl (see below) compared to distribution lines. Therefore, the improvement margin once spirals are attached is greater in transmission lines; and, (iii) larger spirals may be more effective in increasing the visibility of wires [23], [24], reducing collisions to a larger extent.

## Methods

### Study Area

The study was conducted in five important bird areas (IBAs) in central Spain (see [25] for details), which are also the main dry cereal farmland areas in the Madrid region. The terrain is flat to slightly undulating, with a mean elevation of c. 750 m a.s.l. These areas are primarily dedicated to cereal cultivation (mainly wheat *Triticum aestivum* and barley *Hordeum* spp.), with minor fields of legumes *Vicia* spp., grapevines *Vitis vinifera* and olive *Olea europaea* groves. Most cereal is grown in a traditional 2-year rotation system that creates a dynamic mosaic of ploughed, cereal and stubble patches over the region. Small patches of natural vegetation (holm oaks *Quercus ilex*, and scrubland of *Retama* spp. and *Thymus* spp.) remain dispersed across the cereal matrix. Cereal fields are harvested in late June to early July. Stubbles and fallows are also used for sheep grazing [26].

### Study species

We considered all birds that we found dead under the power lines in the study area. We discarded the dead birds found beside poles whose cause of death could be attributed to electrocution. However, since not all species have the same collision risk [2], [8], [9], it is worth noting that the study area holds significant populations of threatened species which are prone to high collision rates due to their low manoeuvrability, high speed flight and/or poor vision [2], [8], [9], such as the great bustard *Otis tarda* (c. 1500 individuals; [27]), little bustard *Tetrax tetrax* (c. 2600 individuals; [28]), pin-tailed sandgrouse *Pterocles alchata* and black-bellied sandgrouse *P. orientalis* (c. 150 and 200 individuals, respectively, [29]).

### Study design and power line monitoring

The study was carried out using a before-after-control-impact (BACI) design, i.e. monitoring power lines before and after the placement of spirals, combined with the use of controls during similar time intervals. Between August 2001 and December 2010 we surveyed bird collisions monthly at 22 different power lines, 7 of them transmission (220 kV) and 15 distribution (15 kV–45 kV) lines, totalling 16.1 and 27.0 km, respectively (Table 1). Fifteen of these lines were our *experimental* lines, i.e. to which spirals were attached. These were monitored once per month for two complete years (one year *before* and one year *after* wire marking). Another 7 lines to which no spirals were attached were used as *control* lines and were monitored also once per month for two complete years. Because no more non-marked control lines were available, in addition to these 7 control lines we also used as controls the second of 10 two-year and the third of 3 three-year surveys carried out at experimental lines once spirals were attached to them (Table 1). These surveys can be considered as *controls* since once the line was marked no changes occurred in the factor presence/absence of spirals and thus no changes were expected between years in the variable under study, i.e. collision rate. The resulting number of power lines (35) and the total length surveyed monthly (72.5 km) for all study years make our study both the most detailed and that with the largest number of power lines monitored to date (for instance, the mean number of power lines per study was 1.9 in a recent review, see Appendix S2 in [19]).

**Table 1:** Power line name, type of line (transmission or distribution), design (experimental or control) and number of years monitored after spiral attachment.

Power line	Type	Length (km)	Design	Times after
Aranjuez E-O	Distribution	2.0	Control	One
Aranjuez N-S I	Transmission	2.0	Experimental	One
Aranjuez N-S II	Transmission	4.1	Experimental	One
Belvis-Cobeña	Transmission	3.0	Experimental	Three
Camarma-Fresno	Distribution	2.0	Experimental	Two
Camarma-Meco	Transmission	1.6	Experimental	Two
Camarma-Torote	Transmission	2.1	Experimental	Three
Campo Real-Valdilecha	Distribution	3.2	Experimental	Two
Daganzo-Alcalá	Distribution	0.9	Control	One
Daganzo-Fresno Rio	Distribution	1.1	Control	One
Daganzo-Torote	Transmission	1.8	Experimental	Three
El Colegio	Distribution	3.0	Experimental	Two
La Cueva-El Casar	Distribution	1.5	Control	One
Mesones	Distribution	2.0	Control	One
Pinto	Transmission	1.5	Experimental	Two
Pozuelo-Valdilecha	Distribution	2.6	Experimental	Two
Quer	Distribution	1.4	Experimental	One
San Martín de la Vega	Distribution	1.7	Experimental	Two
Valdepiélagos-Talamanca I	Distribution	2.2	Experimental	One
Valdepiélagos-Talamanca II	Distribution	0.5	Control	One
Valdetorres-La Jara	Distribution	1.4	Control	One
Villanueva-Quer	Distribution	1.5	Experimental	One

One month before the beginning of each monitoring year we removed all carcasses under the power line. Each monthly search for bird carcasses was carried out by one observer walking at a slow, regular pace parallel to the wires but making zigzags to reasonably visually cover a 25 m band at each side of the vertical of the central conductor wire. The observer surveyed first one side along the line (e.g. the 25 m band on the right side), and then he/she returned to the starting point surveying the other side (25 m band on the left side). All remains found were identified to the species level and removed to avoid double counts. When the species was unknown (<2% of the cases), the carcass was assigned to one of the four sizes considered (see below). We recorded a carcass when the remains found consisted of more than five feathers in a square meter, because a smaller number of feathers cannot safely be interpreted as a collision, since they could have been lost by a bird during preening, moulting or fighting [30]. Carcass searches were not performed in June because crop height may lead to unrealistically low carcass detection figures. July surveys were always carried out after cereal harvesting. However, it is worth noting that in our rather structurally-homogeneous study area, there was no relationship between vegetation height or cover and carcass detection rates [25].

Potential detection biases such as site- or year-dependent carcass removal by scavengers or variation in carcass detection due to habitat heterogeneity are minimized in our study, since we used a BACI design combined with the use of control power lines at the same time intervals. Furthermore, potential outbreaks in scavenger populations are unexpected because predator control is widespread in our study region [31]. However, since monthly search frequencies may be adequate to detect medium- to large-sized corpses, but are insufficient for smaller birds, we used equations from [25] to adjust our mortality estimates in relation to search periodicity and carcass size (Table 2), because both can influence mortality estimates. The correction of field data is important because larger carcasses are detected by researchers more easily than smaller ones, and because the longer time elapsed between consecutive searches and the smaller the size of the carcasses, the larger the effect of scavengers on corpse disappearance [25]. Ideally, surveys to evaluate carcass losses should be carried out in each study area before undertaking further mortality studies [25], because detection rates can differ among study areas (e.g., due to habitat biases, [30]). Therefore, we used our own correction equations instead of others recently published (e.g., [32]). Observers were previously trained in order to minimize potential biases due to their different levels of expertise in carcass searches [25].

**Table 2:** Equations from [25] used in our study to correct numbers of dead birds found at the power line, in order to account for removal by scavengers or missed observations during carcass searches.

Equation	
A_n_ (Detectability)	A_1_ : Large = (no. carcasses found+1)*100/71.7A_2_ : Medium = (no. carcasses found+1)*100/55.8A_3_ : Small = (no. carcasses found+1)*100/32.1A_4_ : Very small = (no. carcasses found+1)*100/33.3
B_n_ (Periodicity and scavenging)	B_1_ : Large = 0.744+28.063*log10(days)B_2_ : Medium = −1.751+41.880*log10(days)B_3_ : Small = −6.623+58.111*log10(days)B_4_ : Very small = 13.538+60.342*log10(days)
C_n_ (Correction)	(A_n_*B_n_)/100
Mortality estimate _n_	A_n_+C_n_

Different equations are given for the four size categories specified in [25] (see Table 3 for their weights). We first corrected the number of carcasses found in the field by their size-dependent detectability (A). Second, we applied equation B for different carcass sizes where “days” is the number of days elapsed from the last visit. Third, we obtained a correction for every size category. Finally, we added C to A to obtain the mortality estimates for each category. The mortality estimate for a given power line was the sum of mortality estimates for the four carcass sizes.

**Figure 1:**
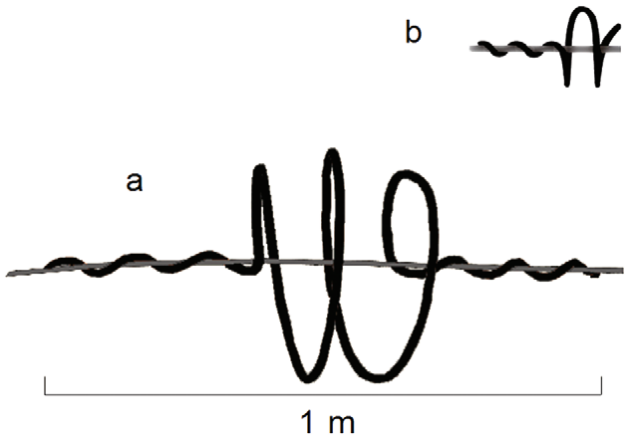
Spirals used in our experiments. Difference in size between large (a) and small (b) can be appreciated.

In addition to testing the effectiveness of line marking as a means to reduce bird collision rate, we also evaluated two potential sources of variation in marking efficiency: power line type and spiral size. Whereas all transmission lines were equipped with large spirals (35 cm diameter and 1 m length, Figure 1a), either large or small spirals (10 cm of diameter and 24 cm m long, Figure 1b) were attached to distribution lines, with the same spiral size attached to all the spans of a given power line. We compared (i) the differences in marking efficiency in transmission *vs.* distribution lines when equipped with large spirals; and (ii) the efficiency of large *vs.* small spirals to reduce bird mortality in distribution lines.

Unfortunately, we have no data on flight frequencies to estimate collision rates associated with our different designs, but in the study of marking effectiveness alone we used the corresponding *controls* to evaluate potential changes in bird mortality associated with changes in bird population densities. Furthermore, power lines of different categories were surveyed in the same study area, minimizing the effect of potential local differences in bird densities.

### Statistical analyses

As a basic first analytical approach we tested whether there was a trend in the number of bird carcasses found after marking the line compared to before marking. This was done considering each power line as a sample unit, and comparing the number of decreases and increases in casualties recorded after marking (in the case of experimental lines), or in the second survey year compared to the first year (in the case of control lines). These comparisons were performed using the two-tailed sign test for small samples [33]. The same test was carried out using the total *estimated* number of dead birds, i.e. after correcting the number of casualties recorded during the field surveys [25]. To confirm the observed trends, we checked the differences in the accumulated numbers of *estimated* deaths before-after marking (first-second year in the case of controls) and experimental lines-control lines by means of a chi-squared test.

As a second approach we used a Generalized Linear Mixed Model (GLMM) of various independent factors on the monthly estimated collision rate, after applying corrections proposed by [25] to the number of carcasses found to account for carcass losses due to removal by scavengers or to being overlooked by observers. For this analysis we considered one month as a time lapse long enough to allow the use of carcass search results in different months as statistically independent. We performed three GLMMs with Poisson error distributions and log link functions. The three analyses shared the same dependent variable, the *estimated* number of dead birds per month, and standardizing per kilometre of power line [30]. They also shared the random factor (power line). The models were fitted by maximizing the log-likelihood using the Laplacian approximation in R-Program 2.11.1 ([34]; *lmer* in *lme4* package). The three analyses were the following: (i) Marking effectiveness alone: We evaluated the effect of wire marking on bird mortality with two fixed factors, ‘Marked *vs.* non-marked’, with two levels, and ‘First survey year *vs.* second survey year’, also with two levels. This analysis includes both lines marked in the second year, but not in the first, and control lines. (ii) Power line type: We explored the effect of the power line type by including a factor with two levels (transmission and distribution) in the sample of power lines marked with large spirals. (iii) Spiral size: We studied the effect of spiral size through a factor with two levels (large and small) in the sample of distribution power lines.

In order to evaluate the importance of correcting for corpse losses, we performed a *sensitivity analysis* with a second group of GLMM tests where the dependent variable was the raw number of carcasses (i.e., those found in the field, without correction per losses) per km per month. All other parameters remained constant. This was only a methodological approach, as all the findings were based on the above-mentioned *estimated* mortality.

Finally, to study the specificity of the patterns found, we re-analyzed our data from a species-specific point of view. However, most of the species did not allow analyzing them with a GLMM procedure because they were not well represented in all the power lines along the study area. We thus proceeded with Wilcoxon paired-sample tests for the three most common species: (i) doves (rock and domestic doves and wood pigeons, all together), (ii) great bustards and (iii) little bustards. We took into account the changes in mortality (first year *vs.* second year) for the whole power line and separating experimental and control lines. We made these species-specific calculations after correcting the number of casualties recorded during the field surveys, i.e., with *estimated* mortality.

## Results

We found 521 carcasses of 45 bird species, 19 of conservation concern (Table 3). Among experimental lines, most showed a decline in mortality after line marking compared to before line marking (11 lines with a decrease, 4 with an increase; *P* = 0.10, two-tailed sign test). The overall decrease in the number of carcasses recorded in the sample of 15 experimental lines was 88 birds (189 birds before marking, 101 birds after marking, 47% reduction in observed casualties). In control lines we did not observe a significant trend (10 lines with a decrease, 5 with an increase, 5 remained constant, *P* = 0.30, two-tailed sign test), with an overall reduction of 20%.

**Table 3:** Species found dead under power lines in the present study and their size following [25]: XS (<50 g), S (50–150 g), M (150–600 g) and L (>600 g).

Species	Size	Carcasses found	SPEC
Cattle Egret *Bubulcus ibis*	L	9	Non-SPEC
White Stork *Ciconia ciconia*	L	24	SPEC 2
Mallard *Anas platyrhynchos*	L	4	Non-SPEC
Shoveler Duck *A. clypeata*	L	1	Non-SPEC
Black Kite *Milvus migrans*	L	2	SPEC 3
Cinereous Vulture *Aegypius monachus*	L	2	SPEC 1
Marsh Harrier *Circus aeruginosus*	L	1	Non-SPEC
Sparrowhawk *Accipiter nisus*	M	1	Non-SPEC
Common Buzzard *Buteo buteo*	L	1	Non-SPEC
Common Kestrel *Falco tinnunculus*	M	6	SPEC 3
Red-legged Partridge *Alectoris rufa*	M	10	SPEC 2
Common Quail *Coturnix coturnix*	S	3	SPEC 3
Common Moorhen *Gallinula chloropus*	M	2	Non-SPEC
Little Bustard *Tetrax tetrax*	L	57	SPEC 1
Great Bustard *Otis tarda*	L	73	SPEC 1
Stone Curlew *Burhinus oedicnemus*	L	12	SPEC 3
Lapwing *Vanellus vanellus*	M	19	Non-SPEC
Black-headed Gull *Larus ridibundus*	L	2	Non-SPEC
Pin-tailed Sandgrouse *Pterocles alchata*	M	6	SPEC 3
Rock/Domestic Dove *Columba livia*	M	130	Non-SPEC
Wood Pigeon *C. palumbus*	M	49	Non-SPEC
Common Swift *Apus apus*	S	1	Non-SPEC
European Roller *Coracias garrulus*	S	4	SPEC 2
Crested Lark *Galerida cristata*	XS	1	SPEC 3
Skylark *Alauda arvensis*	S	14	SPEC 3
Barn Swallow *Hirundo rustica*	XS	1	SPEC 3
Meadow Pipit *Anthus pratensis*	XS	7	Non-SPEC
Robin *Erithacus rubecula*	XS	1	Non-SPEC
Northern Weather *Oenanthe oenanthe*	XS	1	SPEC 3
Blackbird *Turdus merula*	S	1	Non-SPEC
Reed Warbler *Acrocephalus scirpaceus*	XS	1	Non-SPEC
Melodious Warbler *Hippolais polyglotta*	XS	1	Non-SPEC
Subalpine Warbler *Sylvia cantillans*	XS	3	Non-SPEC
Orphean Warbler *S. hortensis*	XS	1	SPEC 3
Blackcap *S. atricapilla*	XS	2	Non-SPEC
Common Chiffchaff *Phylloscopus collybita*	XS	4	Non-SPEC
Willow Warbler *P. trochilus*	XS	3	Non-SPEC
Magpie *Pica pica*	M	28	Non-SPEC
Jackdaw *Corvus monedula*	M	1	Non-SPEC
European Starling *Sturnus vulgaris*	S	1	SPEC 3
Spotless Starling *S. unicolor*	S	8	Non-SPEC
House Sparrow *Passer domesticus*	XS	3	SPEC 3
European Serin *Serinus serinus*	XS	1	Non-SPEC
Linnet *Carduelis cannabina*	XS	3	SPEC 2
Corn Bunting *Emberiza calandra*	XS	7	Non-SPEC
Undetermined medium-sized bird	M	3	—
Undetermined passerine	XS	6	—

Figures are numbers of carcasses found during the whole study period (2001–2010). Note that statistical analyses were made both with raw data and after applying correction equations proposed by [25] to field data shown in this table. The conservation status is based on [43] criteria: ‘SPEC 1’: European species of global conservation concern; ‘SPEC 2’: Species having global populations concentrated in Europe and an unfavourable conservation status in Europe; ‘SPEC 3’: species having global populations not concentrated in Europe but an unfavourable conservation status in Europe; and, ‘Non-SPEC’: species having global populations not concentrated in Europe and a favourable conservation status in Europe.

The 521 dead birds found represent 14,282 estimated bird collisions, an average 8.2 collisions per month and km, after accounting for carcass removal by scavengers and missed observations during surveys. Significantly more experimental lines showed a decrease in the number of estimated casualties after line marking compared to before line marking (12 lines with a decrease, 3 with an increase; *P* = 0.04, two-tailed sign test). The overall difference in the sample of 15 lines was 316 birds (3,300 estimated birds before marking, 2,984 birds after marking, 9.6% reduction in estimated mortality). The control sample did not show significant before-after differences (10 lines with a decrease, 10 with an increase, *P* = 1.0, two-tailed sign test; total estimated casualties: 4,067 before and 3,931 after marking, 3.3% reduction). A chi-squared test with the former data (3,300, 2,984, 4,067 and 3,931) confirmed the difference between experimental and control samples in the reduction of estimated casualties (χ^2^ = 3.90, *P* = 0.048).

**Figure 2:**
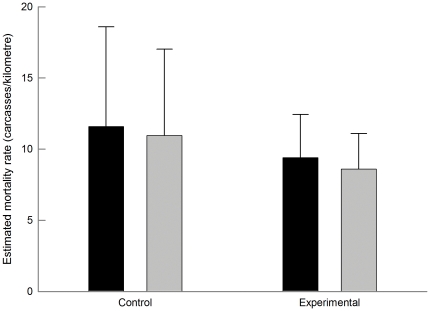
Number of estimated carcasses per kilometre (mean ± SE) before (black) and after (grey bars) marking in control (left) and experimentally marked (right) power lines. Sample sizes were 219 and 165 in each period for control and experimental power lines, respectively.

**Figure 3:**
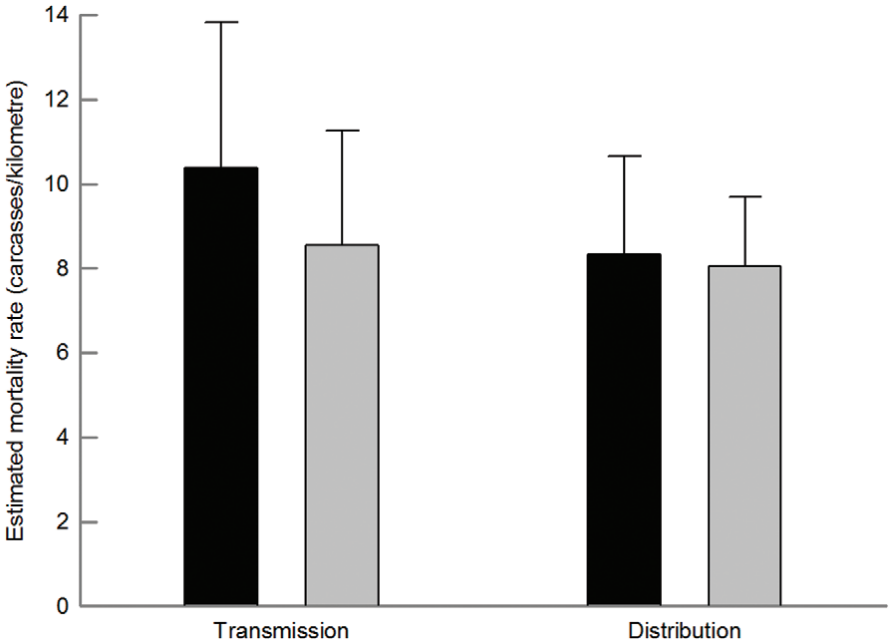
Number of estimated carcasses per kilometre (mean ± SE) before (black) and after (grey bars) marking in transmission (left) and distribution (right) power lines. Sample sizes were 77 and 44 in each period for transmission and distribution power lines, respectively.

**Figure 4:**
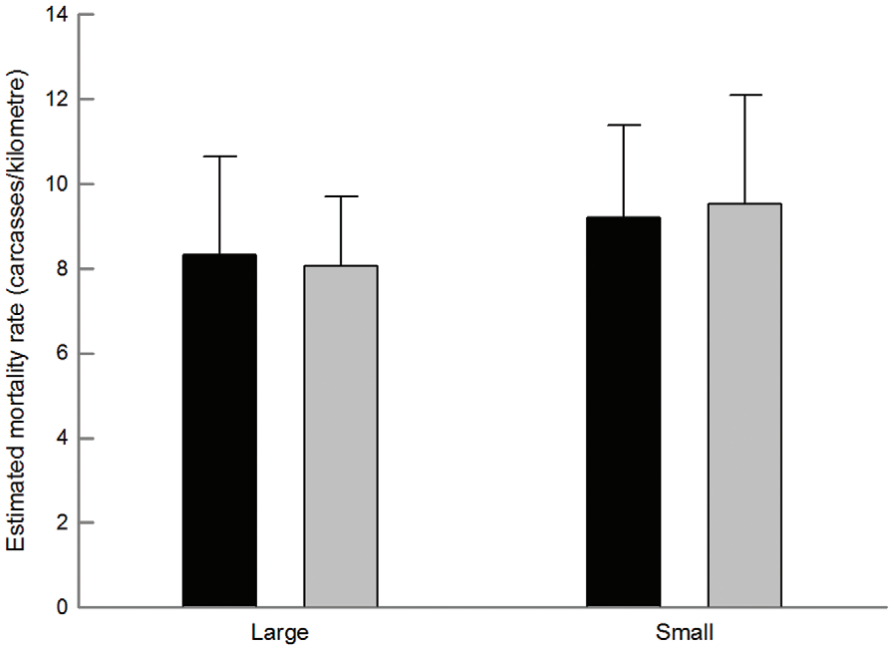
Number of estimated carcasses per kilometre (mean ± SE) before (black) and after (grey bars) marking in distribution power lines marked with large (left) and small (right) spirals. See Figure 1 for more details. Sample sizes were 44 in all cases.

In the GLMM considering all monthly surveys, the number of estimated collisions per kilometre was significantly reduced in experimental power lines after marking, while it remained similar in controls (Table 4i.a; Figure 2). This model explained 96.4% of the deviance. The effectiveness of large spirals was similar in transmission and distribution power lines (Table 4ii.a; Figure 3). The model explained 99.6% of the deviance. Spirals of different sizes had similar marking effectiveness when attached to distribution lines (Table 4iii.a; Figure 4), with 98.8% of the deviance explained by the model. The comparisons with uncorrected raw data (Table 4i.b, ii.b and iii.b) showed different statistical differences (e.g., in ‘marked *vs.* non-marked’), highlighting the importance of correcting field data.

**Table 4:** Parameter estimates from the Generalized Linear Mixed Model for marking effectiveness alone model (i), power line type model (ii) and spiral size model (iii).

(i.a) Marking effectiveness alone (n = 770) (with corrections)				
	Estimate	SE	z	*P*
Intercept	2.34	0.09	27.31	<0.0001
Marked *vs.* non-marked	−0.08	0.04	−2.13	0.03
First survey year *vs.* second survey year	−0.04	0.03	1.57	0.12

(i.b) Marking effectiveness alone (n = 770) (without corrections)				
	Estimate	SE	z	*P*
Intercept	−1.20	0.20	−6.35	<0.0001
Marked *vs.* non-marked	−0.30	0.16	−1.90	0.06
First survey year *vs.* second survey year	0.47	0.14	3.46	<0.0001

(ii.a) Power line type (n = 242) (with corrections)				
	Estimate	SE	z	*P*
Intercept	2.10	0.11	18.49	<0.0001
Power line type	0.11	0.14	0.78	0.44

(ii.b) Power line type (n = 242) (without corrections)				
	Estimate	SE	z	*P*
Intercept	−1.71	0.32	−5.42	<0.0001
Power line type	0.75	0.38	1.99	0.05

(iii.a) Spiral size (n = 176) (with corrections)				
	Estimate	SE	z	*P*
Intercept	2.10	0.08	25.12	<0.0001
Spiral size	0.10	0.12	0.88	0.38

(iii.b) Spiral size (n = 176) (without corrections)				
	Estimate	SE	z	*P*
Intercept	−1.75	0.36	−4.92	<0.0001
Spiral size	0.65	0.49	1.32	0.19

We show GLMM with (a) and without (b) corrections for carcass losses due to researcher overlooking and removing by scavengers. Estimate, standard error (SE), statistic value (z) and statistical significance (*P*) are provided.

Regarding species-specific patterns, doves did not show significant differences in the six treatments, regarding marking effectiveness alone (Wilcoxon paired-sample test, marked *vs.* non-marked, *Z* = 0.87, *P* = 0.39; first survey year *vs.* second survey year, *Z* = 0.00, *P* = 1.00), power line type (transmission lines, *Z* = 0.41, *P* = 0.68; distribution lines, *Z* = 0.41, *P* = 0.68) or spiral size (large spirals, *Z* = −0.32, *P* = 0.75; small spirals, *Z* = −0.50, *P* = 0.62).

In contrast, great bustard mortality was reduced only after marking of transmission lines (transmission lines, *Z* = 2.04, *P* = 0.04; distribution lines, *Z* = 0.00, *P* = 1.00) or only when marking with large spirals (large spirals, *Z* = 2.00, *P* = 0.046; small spirals, *Z* = −0.71, *P* = 0.48), being not significant regarding marking effectiveness alone (marked *vs.* non-marked, *Z* = 1.81, *P* = 0.07; first survey year *vs.* second survey year, *Z* = 0.00, *P* = 1.00).

In the little bustard, wire marking reduced mortality (*Z* = 2.47, *P* = 0.01), whereas statistical differences were not found for controls (*Z* = 0.50, *P* = 0.62) or for power line type (transmission lines, *Z* = 1.79, *P* = 0.07; distribution lines, *Z* = 1.15, *P* = 0.25) or spiral size (large spirals, *Z* = 1.22, *P* = 0.22; small spirals, *Z* = 0.00, *P* = 1.00).

## Discussion

Our results show a slight (overall, 9.6%, after correcting for carcass removal by scavengers and missed observations), but significant reduction in bird mortality after flight diverters were attached to power lines. Regardless of statistical significance, a slight mortality reduction may be very biologically relevant in areas, species or populations of high conservation concern. It is important to note that overall mortality reduction values were not the same if calculated using raw numbers of dead birds found, i.e. before correcting for carcass removal by scavengers and missed observations. This is because correction factors differ between species [25]. Thus, uncorrected mortality values would lead to incorrect conclusions, and special care should be taken when dealing with certain birds of conservation concern. Neither the type of line (transmission *vs.* distribution) marked with large spirals, nor the size of spirals in distribution lines influenced the magnitude of mortality reduction when we assessed overall mortality in all species together. However, great bustard mortality showed reductions when lines were marked with large spirals, and also considering only transmission lines.

The effectiveness of wire marking in reducing bird mortality through collision has been recently reviewed by Barrientos et al. [19]. However, in that study, different markers were combined since available sample sizes did not allow inclusion of marker type as a factor in the analysis. Thus, despite spirals of different sizes and colours being the most frequently employed bird flight diverters, half of the studies included in Barrientos et al. [19] referred to other device types (see Appendix in [19]). The present study suggests that the mortality reduction found in that review was not due to the inclusion of other markers, and that the most widely used spirals are effective. The present study also overcomes a common problem detected in Barrientos et al. [19], namely that sample sizes are generally small. Here we based our conclusions on a large sample including two-year monthly surveys at 15 experimental and 20 control power lines, covering 72.5 km. Moreover, these lines were distributed over a relatively large geographical area, encompassing most farmland areas used by steppe birds in our study region. This overall low (9.6%) reduction could be greater in some places (e.g., migration corridors, power lines close to resting sites, etc), or could represent a valuable reduction for endangered species with high collision risk. Thus, a detailed evaluation of mortality due to collision should be carried out before deciding where to attach spirals as a bird protection measure in relatively large conservation areas.

Some of the species found dead in our study are among those suggested in previous studies to be the most likely to collide with power lines [2], [8], namely those with low maneuverability such as bustards, storks or waterfowl. These species usually fly higher than, for instance, many passerines, and thus most of their collisions are expected to be with transmission lines. Indeed, if we consider the data from the first year only, i.e. before attaching spirals, transmission lines in our study accumulated 71% (n = 42) of all great bustards found dead in all lines, 50% (n = 50) of all little bustards *Tetrax tetrax*, 83% (n = 12) of all white storks *Ciconia ciconia* and 100% (n = 3) of all ducks *Anas* spp., despite the fact that transmission lines represented only 36% of the total length of power lines surveyed. In their study with whooping cranes, Morkill & Anderson [22] found that birds reacted more than expected to transmission lines and less to distribution lines. However, we did not find a significant difference in mortality reduction in marked transmission lines compared to marked distribution lines when we considered all species together. When looking at species-specific patterns, only the great bustard showed a slight mortality reduction in marked transmission lines. Although some studies found that species suffering high collision mortality may show a tendency to avoid areas with transmission lines (e.g. little bustard, [35]), collision with transmission lines is still one of the most important sources of mortality in these species [35], [36]. Thus, as suggested in Barrientos et al. [19], it is possible that at least some of these particularly sensitive species do not properly respond to conventional marking methods (see below).

Although one would expect that large flight diverters are more effective than small diverters in increasing the visibility of marked wires, other authors that have used spirals of different sizes [23], [24] did not statistically test for differences among them. Our study explores this possibility for the first time. Considering all species together, our results suggest that the decrease in collision rate is independent of spiral size, and thus it seems reasonable to conclude that the main advantage of marking is already achieved with small spirals, with larger spirals being unnecessary. This could imply interesting applied findings. For example, small diverters do not apply excessive weight to the wire. Large devices can constitute a problem for this reason especially in high winds, contributing to the downing of power lines, especially if devices are frozen [14], [22]. However, a flagship species like the great bustard showed mortality reduction with larger spirals, suggesting that, at least for this species, large spirals work better.

Despite our study being, to our knowledge, the largest published field experiment, and ca. 310,000 € were spent to mark 33.7 kilometres of power lines in our study area, few conclusions can be drawn beyond the general effectiveness of bird flight diverters in reducing collision mortality. We found differences in effectiveness when we compared markers in transmission versus distribution lines, or when we compared spirals of different sizes in distribution lines only with one species (although we could carry out species-specific analyses only with three species). However, it is worth noting that even after marking, bird collisions in our study area were still high, especially for some endangered species usually showing high collision risks (e.g. great and little bustards). Several non-mutually exclusive explanations could account for this. First, it is possible that the generally low probability of collision (0.21-0.05 birds per 1,000 crossings; [19]) makes it very difficult to find differences even with well-designed experiments. If this is the case, huge experimental designs would be necessary to find larger differences and extract stronger conclusions. Second, it has been argued that bad weather or light conditions can increase bird collisions, especially if birds have problems with flight control [14], [37]. For most birds, sustained slow flight is costly or aerodynamically impossible [38], [39], and hence reducing speed is an unlikely mechanism to increase safety under bad weather or light conditions. Third, collisions frequently occur even under low wind and good visibility conditions [40]. Recent studies [9], [10] suggest that some species, which undergo high collision rates (e.g. bustards and storks) have narrow fields of view in the frontal plane, hindering their ability to see the way ahead. Fourth, Martin [10] suggests that birds flying in open airspace above vegetation could relax –by means of either behavioural or evolutionary adaptations- the monitoring of this airspace since it is a highly predictable environment, usually clear of hazards. In other words, birds of some species could simply not look ahead during flight. Indeed, frontal vision in birds is not a high-resolution vision [10]. Instead, the best resolution occurs in the lateral vision, which most birds employ to detect conspecifics (very important in social species like bustards or storks) and predators, or in identify foraging opportunities. All of these may be more important for a bird than simply looking ahead during flight into open airspace [10]. Fifth, anecdotal events can have potentially important effects on collisions. For instance, Sastre et al. [41] suggest that human-related disturbances causing flight response can increase the probability of collision of great bustards with power lines. Sixth, regarding the effectiveness evaluation of different devices, it is also plausible that misguided approaches have been used to date. For instance, whereas bird flight diverters are usually coloured with a single colour bright to the human eye [19], a recent review [10] recommends the use of black-and-white diverters, which reflect highly or absorb strongly across the full spectrum of ambient light. Thus, it is possible that the few valuable studies carried out to date that compared the effectiveness of different colours for a certain bird flight diverter [42] actually compared colours too close in the spectrum to identify differences in their effectiveness. Since it is recognized that the colour vision of birds extends into the ultraviolet range, thus broadening, compared with humans, the range of stimuli to which the avian eye can respond [10], the use of ultraviolet-devices should be investigated.

In summary of the above-mentioned explanations, and given that is seems clear that no single type of marker will be equally effective for all bird species, we acknowledge that the importance of type and size of bird flight diverters is not yet clear and should be confirmed in future studies. Our study does not pretend to be comprehensive in this respect, and regarding the different susceptibilities of different bird species or groups to collision [see 2,8], and particularly the mortality reductions obtained for specific models of flight diverters should be further investigated. In this sense, we encourage researchers to explore the effectiveness of non-visual diverters. Finally, we highly recommend the identification of mortality hot-spots based on the number of individuals killed and the vulnerability of the species involved [e.g. 44]. Taking into account the economic cost of marking, it is likely more useful to attach flight diverters to these hot-spots rather than to do it to whole sections of power line.
